# The Human Cytomegalovirus UL133-138 Gene Locus Attenuates the Lytic Viral Cycle in Fibroblasts

**DOI:** 10.1371/journal.pone.0120946

**Published:** 2015-03-23

**Authors:** Nirmal Dutta, Philip Lashmit, Jinxiang Yuan, Jeffery Meier, Mark F. Stinski

**Affiliations:** 1 Department of Internal Medicine, University of Iowa, Iowa City, United States of America; 2 Center for Biocatalysis and Bioprocessing, University of Iowa, Iowa City, United States of America; 3 Iowa Veterans Affairs Healthcare System, Iowa City, United States of America; 4 Department of Microbiology, University of Iowa, Iowa City, United States of America; University of Sussex, UNITED KINGDOM

## Abstract

The genomes of HCMV clinical strains (e.g. FIX, TR, PH, etc) contain a 15 kb region that encodes 20 putative ORFs. The region, termed ULb’, is lost after serial passage of virus in human foreskin fibroblast (HFF) cell culture. Compared to clinical strains, laboratory strains replicate faster and to higher titers of infectious virus. We made recombinant viruses with 22, 14, or 7 ORFs deleted from the ULb’ region using FIX and TR as model clinical strains. We also introduced a stop codon into single ORFs between UL133 and UL138 to prevent protein expression. All deletions within ULb’ and all stop codon mutants within the UL133 to UL138 region increased to varying degrees, viral major immediate early RNA and protein, DNA, and cell-free infectious virus compared to the wild type viruses. The wild type viral proteins slowed down the viral replication process along with cell-free infectious virus release from human fibroblast cells.

## Introduction

Human cytomegalovirus (HCMV) is a large, complex DNA virus belonging to the Herpesviridae family. It establishes a lifelong latent infection in the majority of the human population [1]. In individuals with compromised T cell immunity, the virus can reactivate to cause morbidity and mortality [2]. In permissive cells, viral gene expression occurs in three temporal phases, designated immediate-early (IE), early, and late. Transcription of the IE genes is independent of any *de novo* viral protein synthesis. IE gene products activate early viral gene expression, inhibit apoptosis, and neutralize intrinsic and innate cellular immunity (Review in [3, 4]). Early viral proteins participate in viral DNA synthesis. The late genes, which primarily encode structural proteins, are expressed after viral DNA replication.

The major immediate-early (MIE) gene locus, UL122 and UL123, is the most abundantly expressed region under IE conditions and gene products IE1-p72 and IE2-p86 are major factors for determining the fate of the viral replication cycle [3, 5]. There are viral factors associated with the virions that stimulate MIE gene expression, such as tegument protein pp71 and envelope glycoprotein gB [6, 7]. Whether the virions of clinical strains contain additional factors that stimulate or repress MIE gene expression is currently not known.

**Fig 1:**
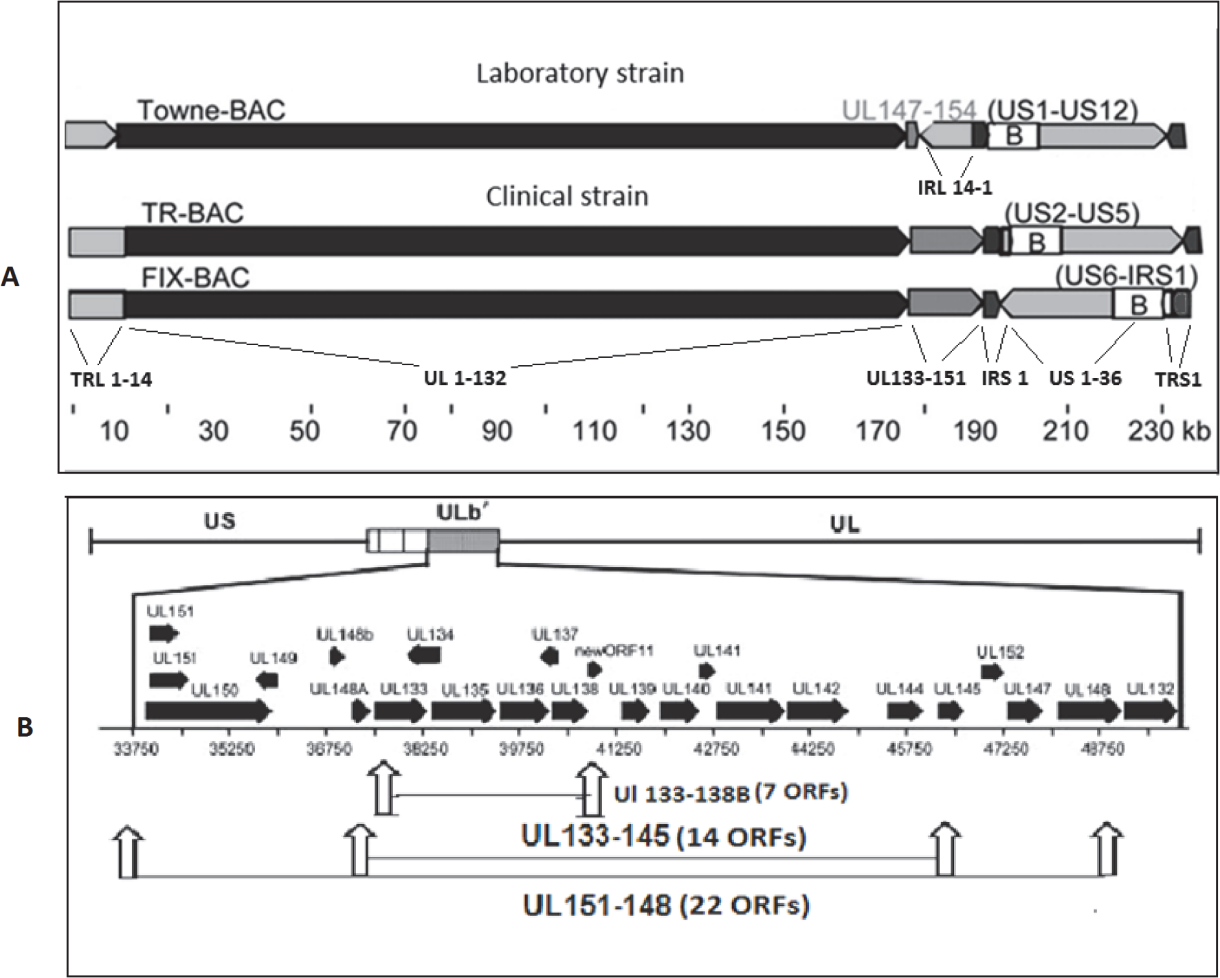
Comparison of ORF organization between HCMV Towne, and two clinical strains. (A) From the left, the Towne genome contains TRL1–14, UL1–132, IRL14–1, IRS1, US1–36 and TRS1. Towne laboratory strain contains a block of ORFs, UL147–UL154. Clinical strains contain RL1–14, UL1–151, IRS1, US1–36, and TRS1. The BAC inserts are identified (B), and viral ORFs deleted during BAC insertions are listed in parentheses (Murphy, et al. (2003) Proc. Natl. Acad. Sci. USA 100, 14976–14981). (B) Predicted ORFs and their direction of expression within ULb’ region were indicated by black arrow as described previously (Petrucelli et al. (2009) J Virol 83, 5615–5629). Schematic representation of ORF deletions to make the mutant recombinant virus are depicted.

The HCMV genome has a coding capacity for hundreds of proteins. Of these proteins, approximately 43 are highly conserved among all members of the herpesvirus subfamilies and are essential for viral replication in cell culture [1]. All clinical strains of HCMV contain a unique region of the genome, termed ULb’, that encodes approximately 20 predicted open reading frames (ORFs) including UL133 to UL151 [8, 9]. The ULb’ region is lost in strains of the virus adapted for replication in cultured human fibroblast cells (Fig. 1). Since these genes are nonessential for viral replication in human fibroblast cells, they are hypothesized to be important for virus dissemination, latency, or pathogenesis in the human host [10, 11]. The cellular and viral mechanisms that determine the balance between latent states and infectious virus replication are best investigated with the clinical strains of HCMV. An understanding of these mechanisms is critical for designing strategies to control HCMV infection and viral pathogenesis.

**Fig 2:**
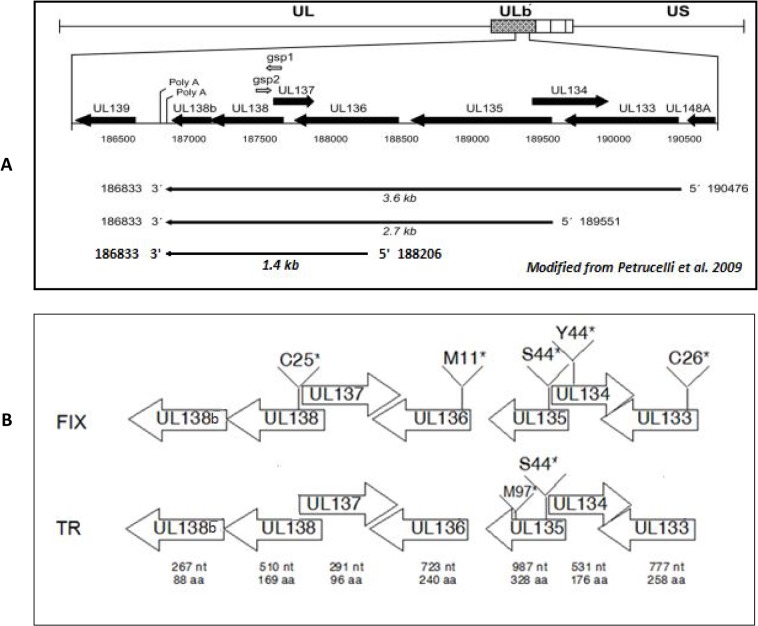
A schematic section of the ULb’ region of recombinant viruses. (A) Predicted ORFs and their direction of expression indicated by black arrows as described previously (Petrucelli A, J Virol. 2009 Jun;83(11):5615–29). (B) Stop codon mutations were introduced into individual ORFs between UL133 and UL138 of clinical strains FIX and TR. Calculated length of individual transcripts in nucleotide (nt) and proteins in amino acid (aa) residues were indicated below each ORF. The amino acid number for the introduction of a stop codon is indicated.

Within the ULb’, the viral protein coding potential studies have concentrated on the UL133 to UL138b region (Fig. 2A). Polycistronic RNAs of 3.6, 2.7, and 1.4 kb are encoded within the region with unique 5’ ends and a common 3’ end [12]. Viral protein expression is determined by protein translation start sites near the 5’ ends of the viral mRNAs and by canonical and non-canonical internal protein translation initiations sites [13]. Within this region, the UL138 ORF has been investigated in more detail. With clinical strains of HCMV (FIX), this viral protein promotes latency in CD34^+^ progenitor cells [10]. With a variant of strain AD169, the UL138 protein increases TNF-α receptors (TNFR-1) [14, 15], which may promote replication in the presence of the cytokine TNF-α. Three additional proteins encoded by UL133, UL135, and UL136 form a viral protein complex with UL138 [13, 16]. These viral proteins are expressed early during productive infection and are detected within the Golgi complex as trans membrane proteins [12, 16]. While these viral proteins promote replication in endothelial cells, they paradoxically retard replication in human CD34^+^ macrophage progenitors and therefore, promote latency [16]. How these viral proteins affect the pathogenesis of HCMV in various cell types is currently not understood and their affects on virus replication are controversial.

We further investigated the affect of the ULb’ region on the efficiency of viral replication in human fibroblast cells by comparing the replication of clinical strains FIX and TR to mutant recombinant viruses with deletions of 22, 14, and 7 ORFs within the ULb’ region (Fig. 1B). All deletions induced more efficient replication in human fibroblast cells, which includes the 7 ORFs between UL133 and UL138b. We also made ORF deletions and stop codon mutations within individual ORFs to determine the affect of the UL133–138b gene cluster on viral replication efficiency. Mutations in these ORFs increased, to varying degrees, all temporal stages of viral RNA and protein, DNA, and infectious virus in the extracellular fluid. The clinical strain ULb’-associated genes slowed down the viral replication process in human fibroblast cells. How these viral gene products might retard the efficiency of viral replication in human fibroblast cells is discussed here.

## Materials and Methods

### Plasmids, BACs, and recombinant viruses

Two recombinant HCMV BAC clinical strains (rHCMV-BAC), FIX-GFP (a gift from Felicia Goodrum, University of Arizona, Tucson, AZ) and TR-GFP (a gift from Dong Yu, Washington University, St. Louis, MO) were used in this study and are referred to as wtFIX-BAC and wtTR-BAC, respectively (Fig. 1A). To delete ORFs or modify a single ORF, the RspL-neo selection and Counter Selection BAC Modification kit (Gene Bridges, Dresden, Germany) was used. The recombination procedure was a modification of Isomura et al. [17] in *E*. *coli* strain DY380 [18]. Oligonucleotide primers (Table 1) were used to amplify a marker cassette containing the neomycin resistance and RpsL (streptomycin sensitive) genes and to simultaneously introduce 50–70 homologous nucleotides at either end of the cassette. Gel purified, linear, double-stranded DNA was subjected to electrophoresis into recombination competent DY380 carrying the targeted rHCMV-BAC DNA for first stage recombination. Intermediate DY380 colonies containing rHCMV with RspL-neo were kanamycin resistant and streptomycin sensitive. To remove the RspL-neo marker cassette, another set of primer pairs with overlapping 3’ ends were used to make a small stretch of double stranded DNA by Pfu DNA polymerase (Life Technologies, Grand Island, NY) extension with oligonucleotides homologous to both ends of the targeted region. Gel purified linear double-stranded DNA was electroporated into recombination competent DY380 carrying the intermediate rHCMV-BAC DNA for second stage recombination. The final form of rHCMV-BAC with the desired deletion was selected on streptomycin containing LB agar plates. FIX-dl 151–148 (22 ORF deletion) and FIX-dl133–145 (17 ORF deletion) were produced using the recombination procedure mentioned above and the primer set listed in Table 1.

**Table 1:** List of oligonucleotides used to construct recombinant FIX and TR viruses.

Name of the primer	Sequence of oligonucleotide (5’—3’)
UL133 F	CGGAGGAAGTTACGTGGGTA
UL138B R	CCAGCTACTGCCGTTAGAGG
UL133 C26* F	ACACCAAATTCCCAAGGCCGCTTAAGTTATCCAAATCACGATGATC
UL133 C26* R	GATCATCGTGATTTGGATAACTTAAGCGGCCTTGGGAATTTGGTGT
UL134 Y44* F	AAGGGCCGGTGTACGGACATTTAAACCTTGGATTTCTGG
UL134 Y44* R	CCAGAAATCCAAGGTTTAAATGTCCGTACACCGGCCCTT
UL135 S44* F	GGCCTCGGCGCTGGGTAAATAATCATATGGCCAGGACC
UL135 S44* R	GGTCCTGGCCATATGATTATTTACCCAGCGCCGAGGCC
UL136 M11* F	CGTCCAAGTCCCACGTCTAGACTGGCATCTCCACGCCC
UL136 M11* R	GGGCGTGGAGATGCCAGTCTAGACGTGGGACTTGGACG
UL138 C25* F	CCAATGGTAAGCTAGATATCAGAGAATGGCCACGAT
UL138 C25* R	ATCGTGGCCATTCTCTGATATCTAGCTTACCATTGG
UL133 RpsL R	GCCGCTGCCGATGGGCGCCGGCGGACGTGACTCGGCAGCCACTGTAGGGATATAGTGCGATGGCGTCAGAAGAACTCGTCAAGAAGGC
UL138B RpsL F	GTTGTGTACATTCGTACTGACAGGGAACCCCCGGTGATGTGCACATTATACTCTTTCATTCTGGGGGGCCTGGTGATGATGGCGGGATCG
UL135 RpsL F	ACGTCGGGGATCTCGAATCGCGCCGGAGGAAACTCGGGTTTATCTATCGGCAGACCATCCTCTCCTGGCCTGGTGATGATGGCGGGATCG
UL135 RpsL R	TAAGGAATTTTCCGACTTGGCCCACATCTCCTTCTTCAGTGTTTGGACAATAAACACATTCCTTGCCTCAGAAGAACTCGTCAAGAAGGC
UL135 F	GAGCAATGCGACGGAGAT
UL135 R	CATCAGACCGGAACGTAACC
UL151-rspl	GTGGCGCCGGAGACACGGGGTGACGTCGGAGACAGGGGCCTTTTGCGGCAGGGACGGGCCTGGTGATGATGGCGGGATCG
UL148-rspl	CGATGAATCTGTCTAGTGACACCAGCCAACCCTCTGCTTTTGCGGGCAAGCGCGCTTCAGAAGAACTCGTCAAGAAGGCG
UL151-Hf	GGGACGGAGGTGGCGCCGGAGACACGGGGTGACGTCGGAGACAGGGGCCTTTTGCGGCAGGGACGAGCGCGCTTGCCCGC
UL148-Hr	TCAGCAATGTGTTGAGGTACTGCACGATGAATCTGTCTAGTGACACCAGCCAACCCTCTGCTTTTGCGGGCAAGCGCGCT
UL133-rspl	TTCAAAGATTCCATAATCGACATTTTAACTTGCCGATGGGTGCGCTACTGCAGCTGGGCCTGGTGATGATGGCGGGATCG
Ul145-rspl	CGGCTGTCACGGCACTGTATCGATGTAACACTAGGGACTTTCTTTGCGATGTAGCCTCAGAAGAACTCGTCAAGAAGGCG
UL-133-Hf	CGGGAATTTTTCAAAGATTCCATAATCGACATTTTAACTTGCCGATGGGTGCGCTACTGCAGCTGGGCTACATCGCAAAG
Ul145-Hr	CAACAGGCATGAGCTGCAGGGCCACGGCTGTCACGGCACTGTATCGATGTAACACTAGGGACTTTCTTTGCGATGTAGCC

The region between UL133 and UL138b was replaced with the RpsL-neo marker cassette to generate FIX-dl133–138b (7 ORF deletion) as well as stop codon mutant recombinant BACs. Primers UL133 RpsLR and UL138b RpsL F (Table 1) were used to amplify the RpsL-neo cassette with ends homologous to UL133 and UL138b by PCR using Platinum PFX Polymerase (Life Technologies, Grand Island, NY). The 1.5 kb PCR product was fractionated on a 0.8% agarose gel in 1X TBE and purified with the Qiagen Gel Extraction Kit (Qiagen). Homologous recombination was performed with 100 ng of PCR product electroporated into DY380 containing wtFIX-BAC or wtTR-BAC DNA to generate the clones FIX-dl133–138b and TR-dl133–138b, respectively.

The RpsL-neo cassette flanked by UL135 sequence was amplified by PCR with pRpsL-neo as template and the primers UL135RpsLF and UL135RpsLR. Homologous recombination with 100 ng of the gel purified PCR product and wtFIX or wtTR in DY380 generated intermediate FIX-dlUL135 and TR-dlUL135, respectively.

To introduce stop codons into respective ORFs, the plasmid pGEM+UL133–138b was generated by amplifying the UL133 to UL138b region of wtFIX-BAC by PCR. Platinum PFX Polymerase (Life Technologies, Grand Island, NY) was used according to the manufacturer’s recommendations with the primers UL133F and UL138bR (Table 1) to produce a 4,457 base pair DNA product. The PCR product was purified with the Qiagen PCR purification kit (Qiagen, Valencia, CA) and then incubated with Taq polymerase (New England Biolabs, Ipswich, MA) at 72°C for 10 min in the presence of 0.2 mM dNTPs to add an adenosine residue to the 3’ end of the DNA. The resulting DNA was cloned into pGEM using the pGEM-T-Easy kit (Promega, Madison,WI) to generate pGEM+UL133–138b. pGEM+UL133–138b was used as template to generate mutations in the genes between UL133 and UL138b with the QuikChange XL kit (Agilent Technologies, Inc. Santa Clara, CA) and the primer pairs listed in Table 1. Plasmids generated were pGEM+UL133–138b UL133 C26*, pGEM+UL133–138b UL134 Y44*, pGEM+UL133–138b UL135 S44*, pGEM+UL133–138b UL136 M11* and pGEM+UL133–138b UL138 C25*. The region containing UL133-UL138b was amplified from the above plasmids using Platinum PFX Polymerase and the primers UL133F and UL138bR. The 4.5 kb PCR product from different plasmid templates was separately fractionated on a 0.8% agarose gel in 1X TBE and purified with the Qiagen Gel Extraction Kit (Qiagen). Homologous recombination was performed with 100 ng of PCR product from each template subjected to electrophoresis into DY380 containing HCMV FIX-BAC-dlUL133–138b to generate FIX-133*, FIX-134*, FIX-135*, FIX-136* and FIX-138* (* indicates the insertion of a stop codon as diagramed in Fig. 2B). To replace the methionine codon at position 97 of UL135 with a stop codon, another round of mutagenesis was performed. The QuikChange Mutagenesis Kit with pGEM+UL133–138b UL135S44* as template and primers UL135 M97* F and UL135 M97* R was used to generate pGEM+UL133–138b UL135S44*M97*. PCR to amplify the UL135 S44*M97* mutation was performed using Platinum PFX Polymerase, pGEM+UL133–138b UL135S44*M97* as template and the primers UL135F and UL135R. The mutation in UL135 at S44 and M97 was introduced into wtTR-BAC by homologous recombination. The 0.7 kb PCR product was fractionated on a 0.8% agarose gel in 1X TBE and purified with the Qiagen Gel Extraction Kit (Qiagen). Homologous recombination was performed with 100 ng of PCR product electroporated into DY380 containing intermediate TR-dlUL135 to generate TR-135* S44, M97.

Reverting the FIX-135* into wild type genome sequence was made by recombination between UL135 PCR product (using primer UL135F and UL135R along with FIX-wt as template) and FIX-135*-BAC DNA in DY380. It was designated as RevFIX-135*. All recombinant HCMV BAC DNAs were digested with the endonuclease HindIII, fractionated in a 0.6% agarose gel, and analyzed for any spurious viral genome deletions or rearrangements. All site-specific mutations in recombinant HCMV BAC DNAs were confirmed by DNA sequencing (University of Iowa DNA Core Facility).

### Recombinant virus isolation

Anonymous samples of human foreskins to prepare primary fibroblast cells were obtained from the University of Iowa Hospital and Clinics. The University of Iowa Hospital and Clinics does not have a consent procedure regarding the storage of human fibroblast from discarded human foreskins. At the time for the start and the completion of this project, there was not a need to obtain informed consent from the University of Iowa Institutional Review Board 01 (Biomedical) for obtaining normally discarded human foreskin tissues that could not be linked to private identifiable information. Also we have had no contact with patients. HFF cells were isolated and grown in Eagle’s minimal essential medium (MEM; Mediatech, Herndon, VA) supplemented with 10% newborn calf serum (Sigma, St. Louis, MO), as described previously [19, 20]. Cells were transfected with either 2 or 5 μg of each recombinant BAC DNA in the presence of 1 μg of plasmid pSVpp71 and 1 μg of salmon sperm DNA (carrier) by the calcium phosphate precipitation method of Graham and Van der Eb [21]. After 5 days, viral cytopathic effect (CPE) appeared. Media was replaced every 3 to 4 days. Seven days after 100% CPE, the extracellular fluid and monolayer of cells were collected. Cell associated virus was released by three freeze and thaw cycles. Cell and supernatant media containing virions were pelleted through a 20% sorbitol cushion by ultracentrifugation as described previously [19]. The virus containing pellet was suspended in 50% newborn calf serum and stored at −80°C. Each virus stock was titrated for plaque forming units (PFU/ml) using a GFP-based plaque assay method on HFF cells with agar overlay as described previously [22].

### Western blotting

Cells were harvested, lysed, and processed for Western blot analysis as described previously [23, 24]. Briefly, cell lysates were fractionated by SDS-PAGE in a 4 to 15% gradient gel. Proteins were transferred to polyvinylidene difluoride (PVDF) membranes, and the following antibodies (Ab) were used: anti-MIE monoclonal Ab (MAb) 810 (Chemicon, Temecula, CA), anti-UL99 MAb (Fitzgerald, Concord, MA), anti-pp65 MAb (Fitzgerald), anti-pp71 MAb (Acris, Herford, Germany) and anti-glyceraldehyde3-phosphate dehydrogenase (anti-GAPDH; Chemicon), Proteins were detected using secondary horseradish peroxidase (HRP)-conjugated goat anti-mouse immunoglobulin G (IgG) and SuperSignal West Pico chemiluminescence detection reagent (Pierce, Rockford, IL) according to the manufacturer’s instructions. To determine equal amounts of infectious virus input, virion tegument protein pp65 was assayed by Western blot analysis at 3 hours post infection (h p.i.). Rabbit polyclonal antibodies to UL133 and UL135 were a kind gift from F. Goodrum (Univ. of Arizona). Image J program was used to calculate the band intensity and the ratio of corresponding proteins from scan pictures of equally exposed films.

### Real-time PCR analysis

HCMV gB DNA was detected and quantified using multiplex real-time PCR for either viral DNA input or viral DNA synthesis. Whole-cell DNA was harvested at 3 h p.i. for viral DNA input or at various days post infection (d p.i.) for viral DNA synthesis assay as described previously [23, 24]. Real-time PCR was performed using 0.4 μg DNA in a final volume of 20 μl of the Platinum PCR Supermix-UDG cocktail (Invitrogen, Carlsbad, CA). HCMV gB primers and 6-carboxyfluorescein-6-carboxytetramethylrhodamine probe, and cellular 18S DNA primers and VIC-6-carboxytetramethylrhodamine probe (Applied Biosystems, Foster City, CA) were used simultaneously. Thermal cycling conditions and quantification of gB DNA were as described previously [25]. The relative amount of input viral DNA was expressed relative to either wtFIX, RevFIX-135*, or wtTR in three respective sets of experiments.

HCMV MIE, IE1, IE2 and cellular 18S RNAs were detected and quantified using real-time PCR. HFF cells were infected in triplicate with recombinant virus wtFIX, FIX-133*, FIX-135*, FIX-136*, FIX-138* and FIX-dl133–138b at an MOI of 0.5. After 3, 18, 24, 36 and 48 h, total RNAs were isolated using TRIreagent (Invitrogen) and treated with RNase-free DNase. 1 μg of total RNA was converted to cDNA using SUPERSCRIPT II RNase H-negative reverse transcriptase (RT) (Life Technologies, Gaithersburg, Md) according to the manufacturer protocol. Multiplex real-time PCR was performed using 2 μl of 1:5 diluted cDNA in a final volume of 20 μl of the Platinum PCR SuperMix-UDG cocktail (Invitrogen). Primers and probes for HCMV MIE, IE1, and IE2, genes were described previously [26, 27]. The primers and the VIC-labeled probe used to amplify the ribosomal 18S were obtained from PE Applied Biosystems (Branchburg, NJ). Viral MIE, IE1, and IE2 RNA levels were normalized to cellular 18S RNA and the values for wild-type virus at 3 h p.i. were set as 1.

### Immunofluorescence assay

HFF cells in 24 multiwell plates with a coverslip on the bottom were infected with wt or mutant recombinant viruses at an MOI of 0.5 and harvested for immunofluorescence assay at 24 h p.i. as described previously [28]. Cells were examined and photographed with an Olympus DX-51 fluorescence microscope. The primary antibody was anti-IE1-p72 (Chemicon International) and the secondary antibody was goat anti-mouse antibody conjugated to Alexa Fluor 555 (Molecular Probes Invitrogen, Eugene, OR).

## Results

### Effect of ULb’-associated gene deletion on MIE protein expression

**Fig 3:**
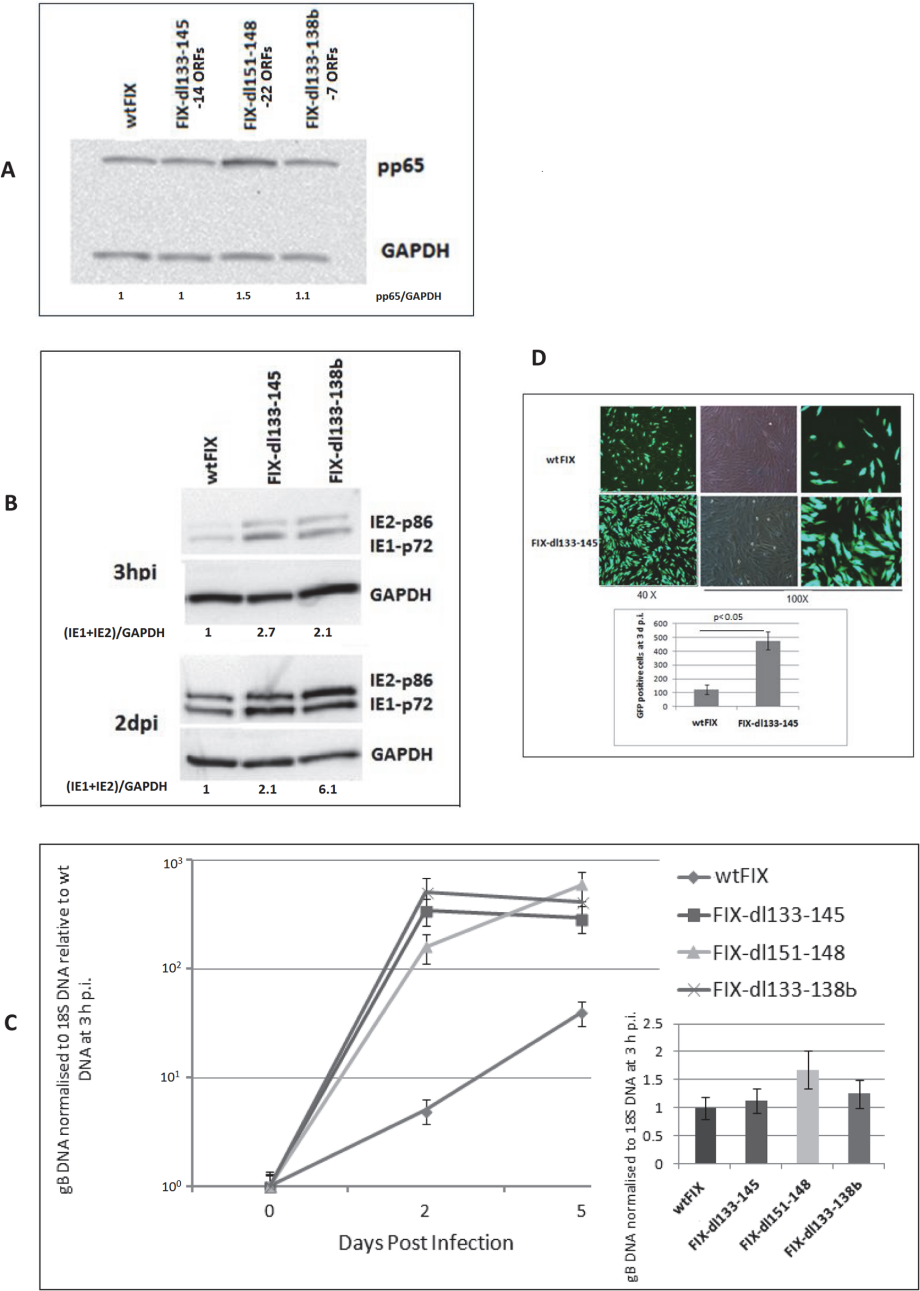
Effect of viral gene deletions in the ULb’ region. Three recombinant viruses with ORF deletions from 7 ORFs to 22 ORFs are designated. HFF cells were infected with wt and recombinant viruses at an MOI of 0.5 PFU/cell and a separate batch of cells were harvested for both protein and DNA analysis at various times after infection as described in Materials and Methods. (A) Western blot analysis of the pp65 protein associated with the infected cells at 3 h p.i. to demonstrate equivalent virus particle input. GAPDH was used as a loading control. (B) Western blot analysis of MIE protein at various times after infection. (C) Quantification of gB DNA normalized to 18S DNA using Real Time PCR at various d p.i. The viral DNA input at 3 h p.i. is in the insert. (D) GFP fluorescence or bright field microscopy of cells infected with wtFIX virus genome modified with a GFP expression cassette or recombinant virus FIX-dl133–145 at 3 d p.i. Average number of GFP positive cells were counted from 6 independent fields using ImageJ program and plotted in a bar graph.

Clinical HCMV strains, like FIX and TR, have additional ORFs between UL133 and UL151 when compared to laboratory strains Towne and AD169 [9]. During repeated passage of the Towne strain in human fibroblast cells, part of the genome was deleted and the terminal repeat was duplicated (Fig. 1A). Since clinical strains like FIX and TR replicate slowly in HFF cells compared to Towne, we investigated whether the clinical gene products have an affect on viral MIE gene expression. We deleted 22 ORFs from wtFIX-BAC to generate FIX-dl151–148 using a RspL-neo two step recombination procedure as described in Materials and Methods. To narrow down the target sequences, two additional deletion mutant recombinant FIX viruses, FIX-dl133–145 (14 ORFs) and FIX-dl133–138b (7 ORFs), were also constructed (Fig. 1B). HFF cells were infected with the above recombinant mutant viruses at an MOI of 0.5 PFU/ cell. After washing the cell monolayer three times with PBS, pH 7.4, the cells were harvested at 3 h p.i. for either Western blot analysis of tegument protein pp65 or for quantitative PCR for viral DNA to demonstrate equal virion input. Fig. 3A shows that approximately the same amount of pp65 protein was associated with the virus-infected cells at 3 h p.i. FIX-dl133–145 and FIX-dl133–138b had qualitatively higher levels of IE1-p72 and IE2-p86 at 3 h p.i. and at 2 d p.i. (Fig. 3B). In addition, approximately the same amount of viral DNA (gB DNA) was associated with the virus-infected cells at 3 h p.i. (Fig. 3C insert). This indicates that the steady state levels of MIE protein was higher when the ULb’-associated genes were deleted.

### Effect of deleting the ULb’-associated genes on viral DNA replication and growth

To determine what effect the ULb’-associated genes had on viral replication, HFF cells were infected with wtFIX, FIX-dl151–148, FIX-dl133–145 and FIX-dl133–138b at an MOI of 0.5 PFU/cell. We analyzed the total amount of viral gB DNA at 3 h p.i. (Fig. 3C insert) and after various times of infection. gB DNA was normalized to cellular 18S DNA and was made relative to wtFIX gB DNA at 3 h p.i. FIX-dl151–148, FIX-dl133–145 and FIX-dl133–138b produced approximately 10- to 60-fold more viral DNA than wtFIX at 5 d p.i. (Fig. 3C). To determine the proportion of cells with an active infection, we used expression of the GFP gene recombined into the viral genome as described previously [9] and counted the number of green fluorescence cells from 10 independent fields using ImageJ software. Fig. 3D compares wtFIX and FIX-dl133–145 at 3 d p.i. at both 40X and 100X magnification. FIX-dl133–145 had approximately 4-fold more green fluorescent cells than wtFIX. Higher MIE gene expression with the mutant recombinant viruses correlated with higher viral DNA replication and infectious virus growth relative to wtFIX.

### Effect of stop codon mutation of individual ORFs between UL133 to UL138

**Fig 4:**
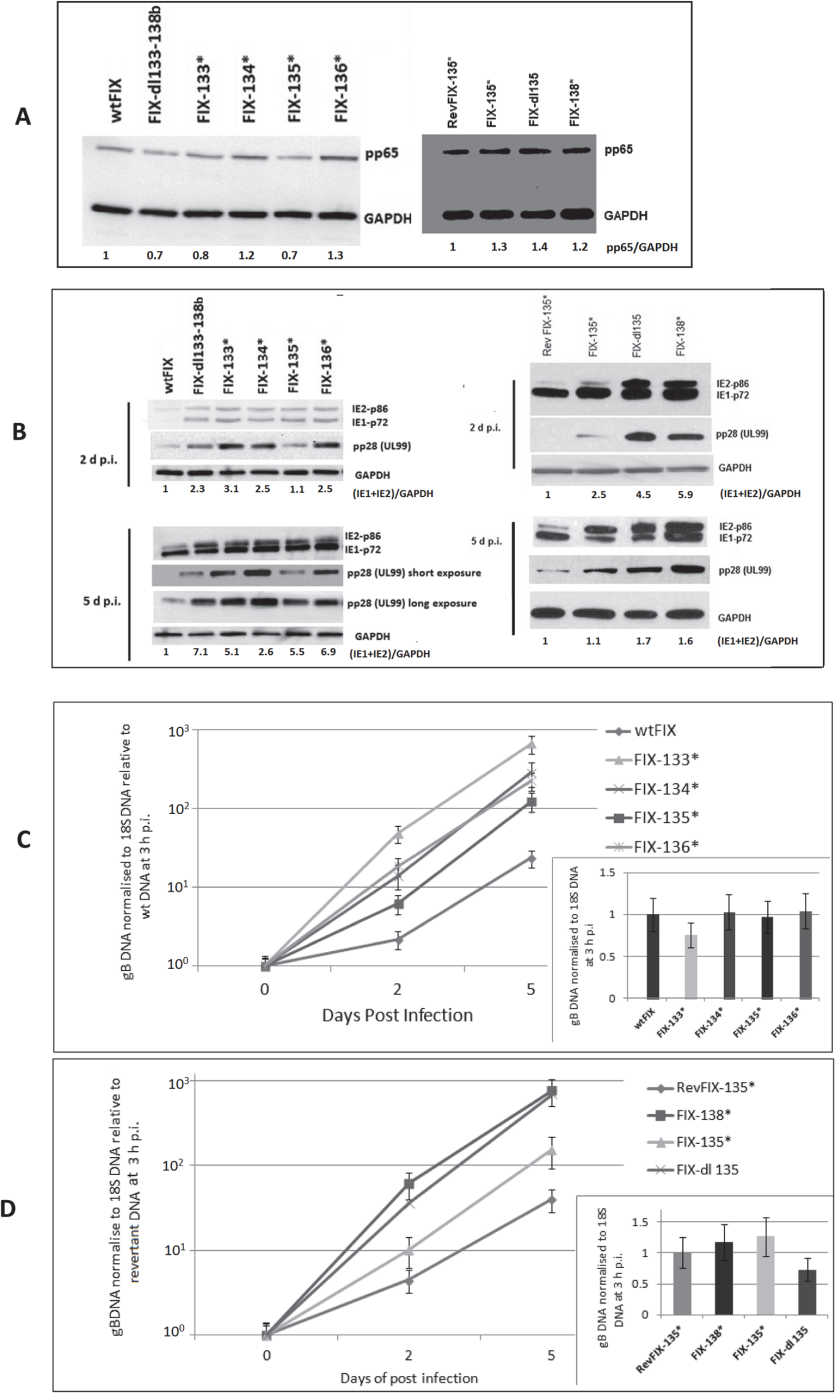
Effect of viral gene deletion and ORF mutation in the UL133–138b region on viral protein and DNA. HFF primary cells were infected with wt and recombinant FIX virus with ORF mutations at an MOI of 0.5 PFU/cell and a separate batch of cells were harvested for both viral protein and DNA analysis. (A) Western blot analysis to determine equivalent virus particle input by quantifying the pp65 protein associated with the infected cell at 3 h p.i. (B) Western blot analysis of MIE and late viral protein. GAPDH served as a loading control. (C and D) Quantification of gB DNA normalized to 18S DNA using real time PCR. The insert shows equal viral DNA input at 3 h p.i.

To determine whether it was simply the removal of viral DNA from the viral genome or absence of a particular protein that facilitated viral replication, we introduced stop codons into each individual ORF into the smallest region (7 ORFs) of UL133 to UL138. Fig. 2A shows that the gene locus has at least three transcription start sites with a common transcription stop site downstream of UL138b [12]. We introduced stop codons into each ORF so as to not alter the expression of overlapping or opposite directional ORFs. Schematic representation of the ORFs along with the position of stop codon mutations in mutant recombinant FIX virus are shown in Fig. 2B. These viral proteins have been demonstrated by Western blot analysis at higher MOIs [16, 29]. Western blot analysis of the mutant recombinant virus-infected HFF cells using polyclonal antibody against UL133 and UL135 showed the absence of the corresponding protein (S1 Fig.). UL135 may have expressed shorter length polypeptides from downstream Met codons. HFF cells were infected with either wtFIX or revertant (Rev)FIX-135* and the mutant recombinant viruses at an MOI of 0.5 PFU/cell. To prove equal amount of infectious virus input, we assayed for viral tegument protein pp65 and viral gB DNA at 3 h p.i. Fig. 4A shows that the HFF cells had the same input of viral tegument protein pp65 for the wt or revertant viruses and the various mutant recombinant viruses. All mutant recombinant viruses had qualitatively higher steady state levels of viral MIE protein and late protein pp28 (UL99) than wtFIX or RevFIX-135* (Fig. 4B). FIX-135* had a relatively lower steady state level of pp28 compared to FIX-133*, FIX-134*, FIX-136* and FIX-138* at both 2 and 5 d p.i. (Fig. 4B). In addition, the inserts to Fig. 4C and 4D show that the same amount of viral DNA was associated with the virus-infected cells.

We also determined the amount of viral DNA (gB DNA) by real time PCR. The amount of gB DNA was expressed relative to either wtFIX or RevFIX-135*. Viral DNA replication was 5 to 30 fold more efficient for stop codon and deletion mutations (Fig. 4C and 4D). However, the stop codon mutation for FIX-135* had less of an effect than the deletion mutation for FIX-dl135 (Fig. 4D). These data indicated that the proteins of UL133 to UL138 affect viral protein and DNA levels in HCMV-infected HFF cells.

**Fig 5:**
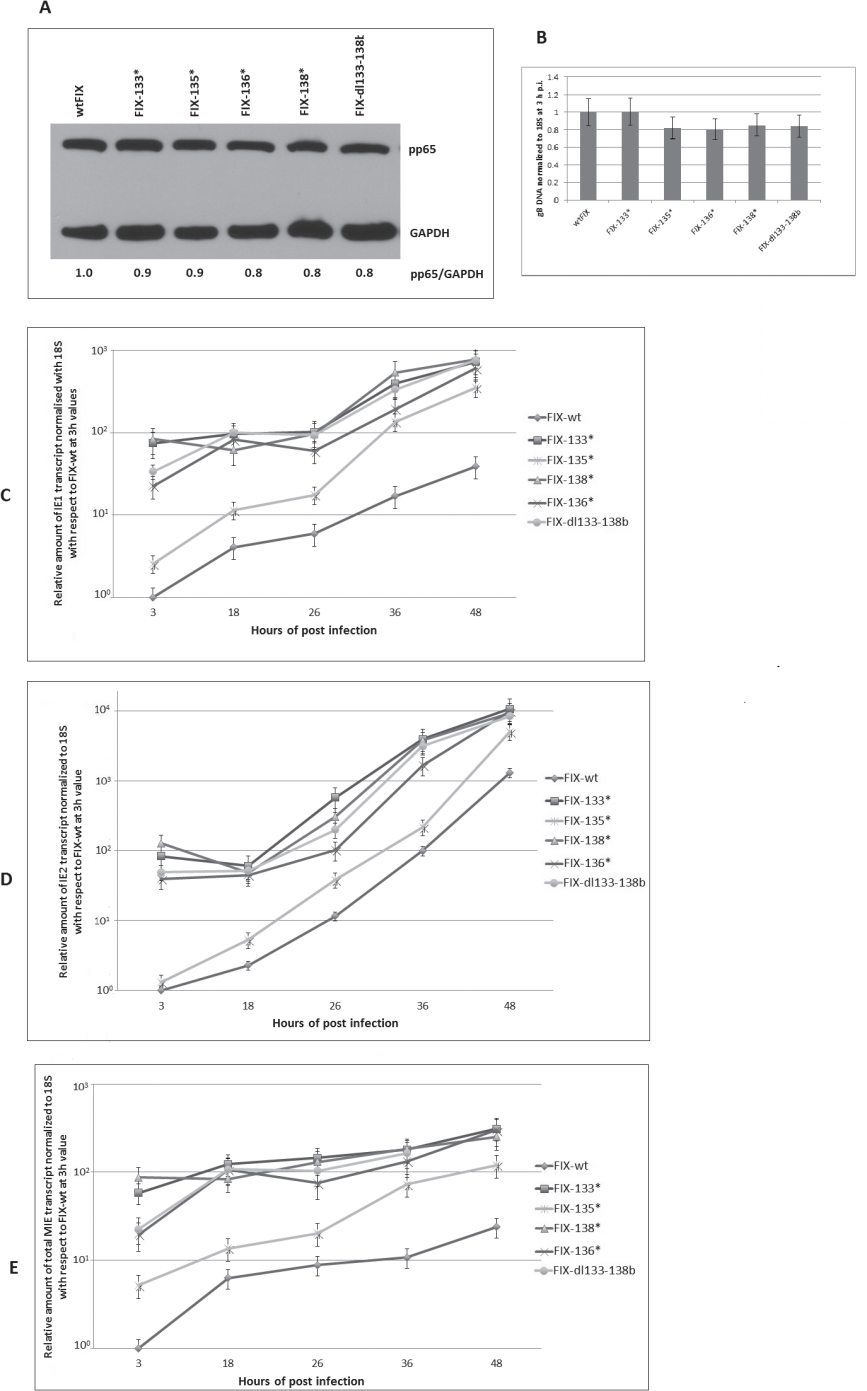
Effect of viral gene deletion and ORF mutation in the UL133–138b region on MIE transcript. HFF primary cells were infected with wt and recombinant FIX virus with ORF mutations at an MOI of 0.5 PFU/cell and a separate batch of cells were harvested for both viral protein and DNA analysis. (A) Western blot analysis to determine equivalent virus particle input by quantifying the pp65 protein associated with the infected cell at 3 h p.i. (B) Quantification of gB normalized to 18S DNA using real time PCR to show equal viral DNA input at 3 h p.i. (C, D, and E) RT-PCR to show IE1, IE2, and MIE RNA at various times after infection with wt and mutant recombinant viruses, respectively.

We also determined the relative amount of MIE RNA using three different sets of primer and probe mix to identify the IE1, IE2 and total MIE transcripts in a different set of experiments. HFF cells were infected with wtFIX and the mutant recombinant viruses at an MOI of 0.5 PFU/cell. To prove equal amount of infectious virus input, we assayed for viral tegument protein pp65 and viral gB DNA at 3 h p.i. Fig. 5A shows that the HFF cells had the same input of viral tegument protein pp65 for the wt and the various mutant recombinant viruses. In addition, Fig. 5B showed that the same amount of viral DNA was associated with the virus-infected cells. The relative amount of IE1, IE2 and total MIE transcripts was plotted in Fig. 5C, 5D and 5E respectively. All stop codon mutant recombinant viruses along with the deletion mutant had higher steady state levels of all MIE transcripts compare to wtFIX at 3 to 48 h p.i. FIX-135* had a relatively lower level of all three transcripts compared to FIX-133*, FIX-134*, FIX-136* and FIX-138*. The relative steady state levels of MIE RNA correlated with the relative levels of MIE protein.

To determine whether a higher relative level of pp71 affected the relative amount of MIE RNA and protein, we determined the amount of cell-associated pp71 as well as pp65 at 3 h p.i. S2A Fig. shows that with an equal MOI of 0.5 PFU/cell, there was an equal relative amount of pp71 protein for wtFIX and the various recombinant viruses. S2B Fig. shows the ratio between pp71 and pp65 normalized to GAPDH from two biologically separate experiments analyzed on three independent Western blots. There were no statistically significant differences (P<0.5) between wtFIX and the various recombinant viruses. These data indicate that changes in the level of pp71 were not related to changes in the level of MIE gene expression. To determine whether an increase in steady state MIE protein was due to more cells expressing MIE protein or more MIE protein being produced per cell, we did immunofluorescence assay for IE1-p72 antigen at 24 h p.i. as described in the Materials and Methods. S3 Fig. shows more IE1 antigen in the infected cell nuclei with the mutant recombinant virus-infected cells than with wt FIX. We conclude that with approximately equal virion input (pp65) and viral DNA input (gB), the wt proteins encoded by UL133 to UL138 suppress MIE gene expression in HFF cells.

### Infectious virus production with the different recombinant FIX viruses

**Fig 6:**
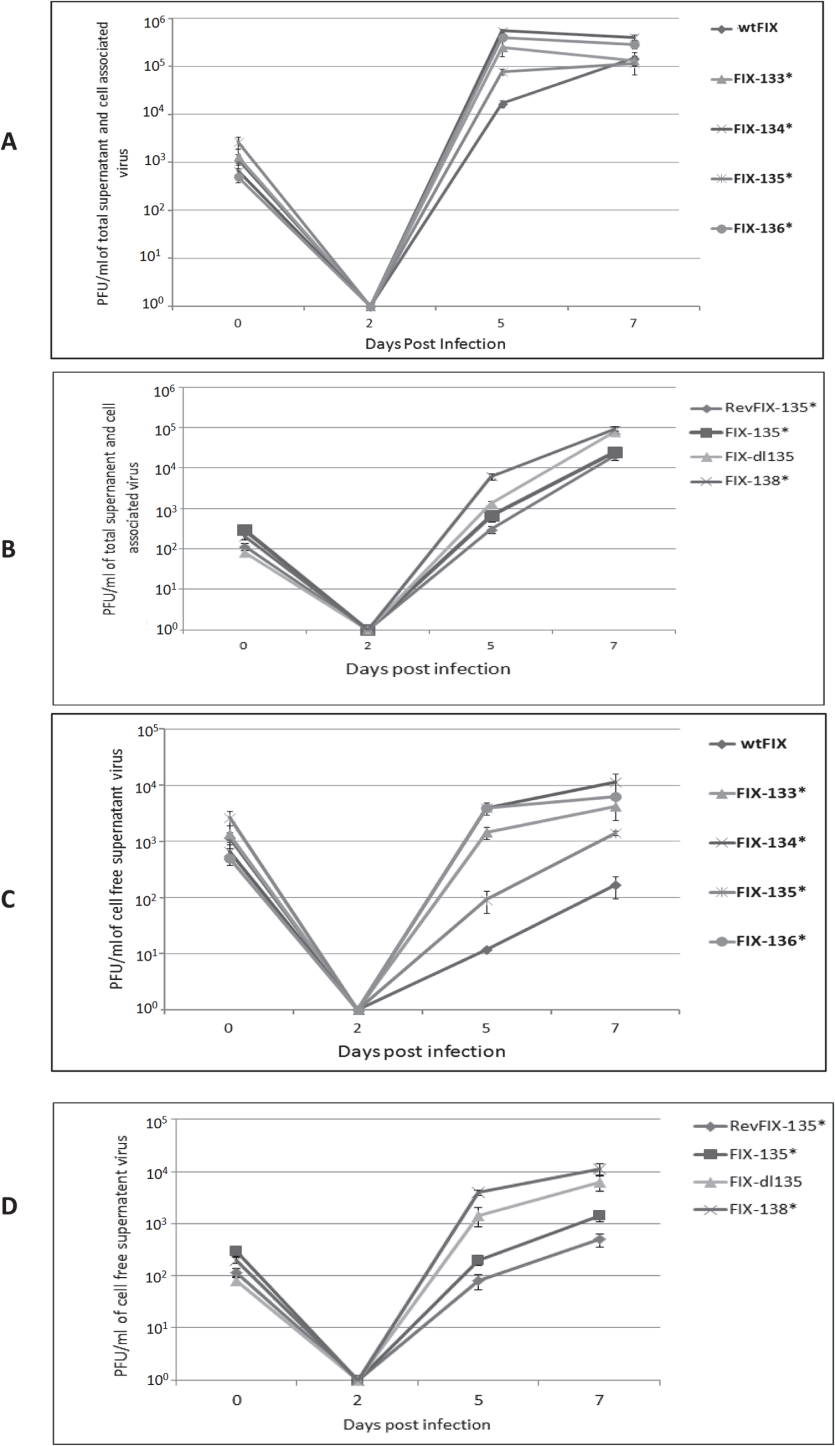
Growth curve analysis of wtFIX and recombinant viruses with ORF mutations in the UL133–138b region. HFF cells were infected at an MOI of 0.5 PFU/cell. (A and B) Total virus at various d p.i. Cells and supernatant were harvested from individual wells and subjected three times to freeze thaw lyses. (C and D) Extracellular virus at various d p.i. Cell free supernatant was collected from individual wells.

To determine if the various mutant recombinant viruses produced more infectious virus than wtFIX or RevFIX135*, we infected HFF cells at an MOI of 0.5 PFU/cell. We determined the titer of cell-associated plus extracellular virus (Total virus) or the infectious virus in the cell-free supernatant. At 5 d p.i., there was approximately 10-fold more infectious virus with the various mutant recombinant viruses than with wtFIX or RevFIX-135*. However, by 7 d p.i., total virus production was relatively similar (Fig. 6A and 6B). In contrast, there were 10- to 100-fold more infectious mutant recombinant viruses in the cell- free supernatant by 5 and 7 d p.i. than wtFIX or RevFIX-135* (Fig. 6C and 6D). FIX-135* infectious virus titers were higher compared to wtFIX or Rev-FIX-135*, but lower compared to the other mutant recombinant viruses with ORF stop codons (Fig. 6C and 6D). These data indicate that the viral proteins encoded by UL133 to UL138 slow down the rate of infectious virus production in HFF cells, which is particularly demonstrable in the amount of infectious virus that accumulates in the extracellular fluid.

### Stop codon mutations and deletions in the UL133–138b locus in HCMV TR

**Fig 7:**
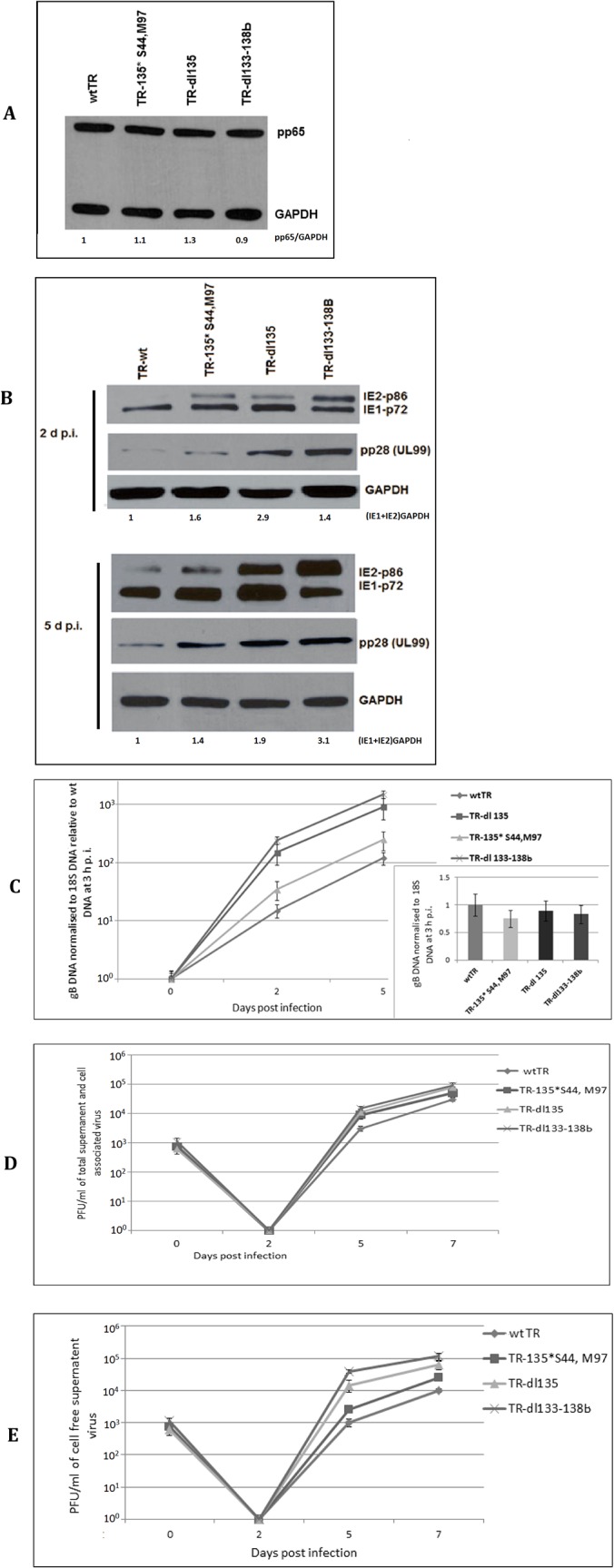
Effect of viral gene deletion and mutation in the UL133–138b region on recombinant TR viruses. HFF cells were infected with recombinant wt and mutant TR virus at an MOI of 0.5 PFU/cell. Separate batches of cells were harvested for both protein and DNA analysis. (A) Western blot analysis to determine equivalent virus particle input by quantifying the pp65 protein associated with the infected cells at 3 h p.i. (B) Western blot analysis of MIE and late viral protein. GAPDH served as a loading control. (C) Quantification of gB DNA normalized to 18S DNA using real time PCR. The insert shows equal viral DNA input at 3 h p.i. (D) Total virus at various d p.i. Cells and supernatant were harvested and subjected three times to freeze and thaw lyses. (E) Extracellular virus at various d p.i.

We selected another HCMV clinical isolate, TR-BAC (Fig. 1A), to substantiate the observations with FIX-BAC. To further investigate the role of the UL135 ORF, we constructed a double stop codon mutation in UL135 ORF (TR-135* S44, M97) (Fig. 2B) as described in the Materials and Methods. The rate of wtTR replication in HFF cells was compared to TR-dl133–138b, TR-dl135, and TR-135*S44, M97. We established equal infectious virus input by determining the amount of tegument protein pp65 and gB DNA associated with the virus-infected cells at 3 h p.i. (Fig. 7A insert and 7C insert, respectively). With the mutant recombinant viruses, IE1-p72 and IE2-p86 and late viral protein pp28 (UL99) had higher relative levels at 2 and 5 d p.i. than wtTR (Fig. 7B). Like FIX-135* (see Fig. 4B), TR-135* S44,M97 had intermediate levels of IE1-p72 and IE2-p86 (5 d p.i.) and pp28 (2 and 5 d p.i.), whereas TR-dl135 had higher levels like FIX-dl135 (Fig. 7B). In addition, viral DNA replication was 2- to 10-fold higher with the mutant recombinant viruses relative to wtTR (Fig. 7C). While a difference in total virus production between wild type and the various recombinant viruses was detected at 5 d p.i (Fig. 7D), there was 10- to 50-fold more infectious virus in the cell-free supernatant with the mutant recombinant viruses relative to wtTR (Fig. 7E). There was also significantly more infectious virus in the cell free supernatant with TR-dl135 and TR-dl133–138b than with TR-135* S44, M97 (Fig. 7E). These data with wtTR and the various mutant recombinant TR viruses confirm the observations in Figs. 2, 4, and 5 with wtFIX and the various mutant recombinant viruses. In addition, the replication of TR-135*S44, M97 was similar to FIX-135*. It is possible that the deletion of UL135 may have affected the expression of UL136 to UL138 by altering the transcription start site of the 2.7 kb RNA (see Fig. 2A). The UL135 mutations suggested that UL135 is important in slowing down HCMV clinical virus replication, but to a lesser extent than UL133, 134, 136, and 138.

Taken together, the mutant recombinant FIX and TR viruses demonstrate that the protein products of the ULb’-associated genes, particularly the region between UL133 and UL138b, slowed down the replication of the virus in HFF cells at all levels, i.e. MIE RNA and protein, late viral proteins, viral DNA, and infectious virus release.

## Discussion

The ULb’ region is nonessential for replication of laboratory strains Towne and AD169 in HFF cell culture. The coding potential of ULb’ region has been shown for eight of the 20 putative ORFs. These include UL133, 135, 136, 138, 141, 142, 144 and 146 [10, 16]. Genes in the ULb’ region likely relate to viral adaptations necessary for latent and persistent infection of the host and immune evasion. Using a recombination mediated deletion procedure, we found that the ULb’ region modulates the kinetics of HCMV temporal gene expression. Deletions within the ULb’ region of 22, 14, and 7 ORFs increased MIE viral gene expression, viral DNA replication, and infectious virus production. To identify the viral ORFs that affect the efficiency of viral replication, we focused on the 7 ORFs between UL133 and UL138b. Using mutant recombinant viruses of FIX or TR clinical isolates, we demonstrated that deletion of UL133 to UL138 up-regulated IE1-p72 and IE2-p86 steady state levels of protein. As expected, viral DNA replication and late viral protein steady state levels were also up-regulated. We tried to identify this function to a single ORF by making stop codon mutations in individual ORFs between UL133 and UL138. However, all stop codon mutations within this gene cluster up-regulated MIE RNA, MIE protein, viral DNA synthesis, and late protein expression. UL133, 136, and 138 affected viral RNA, protein, and DNA synthesis in a very similar pattern, that correlates with these viral proteins being in a protein complex [16]. The UL135 stop codon mutations affected viral RNA and protein and DNA synthesis at a level intermediate between wtFIX and the other stop codon mutations (see Fig. 4). Single and double stop codon mutations within UL135 with FIX or TR, respectively, gave the same results. The double stop codon mutation does not eliminate the possibility of UL135 gene expression due to the remaining downstream ORF. However, expression of the remaining downstream ORF was not observed in HCMV genome wide protein coding potential scanning using the ribosome profiling method [30]. In contrast, the mutant recombinant virus with a deletion of UL135, behave like the other deletion and stop codon mutations (see Fig. 4). Since these genes are expressed from the 2.7 kb polycistronic mRNA (see Fig. 2), the deletion of UL135 might have affected the 5’-end of the mRNA and hampered the expression of the downstream UL136 to UL138 genes. These data suggest that UL135 may have a regulatory function that affects the UL133, UL134, UL136, and UL138 protein complex formation. Further investigation is necessary to understand how UL135 affects the other proteins within the gene cluster.

The increase in viral MIE RNA and protein, viral DNA, and late viral protein, also increased cell-free infectious virus production. When we assayed for total cell-associated plus cell free infectious virus, there were differences between wtFIX and wtTR and the various mutant recombinant viruses at 5 d p.i., but little to no difference by 7 d p.i. (see Fig. 5). Similar results were reported by Umashankar et al. [16] comparing wtFIX or wtTB40E with mutant recombinant viruses using the 50% tissue culture infective dose (TCID_50)_ assay. When we assayed for cell free infectious virus only, there were 10- to 100-fold more mutant recombinant viruses at 5 and 7 d p.i. than with wtFIX or wtTR. These data indicate that the UL133 to UL138 complex of proteins also affects viral egress as well as steady state levels of viral MIE protein.

To demonstrate equal virus input of the different mutant recombinant viruses and the wt virus or revertant virus, we assayed for tegument protein pp65 and viral DNA at 3 h p.i. Regardless of the clinical strain, the mutant recombinant viruses expressed qualitatively higher levels of MIE proteins IE1-p72 and IE2-p86 than the wt viruses. However, we could not attribute these results to differences in the relative levels of virion-associated protein pp71. While differences in the virions or viral nucleocapsids could explain these results, it is unlikely that these viral proteins affect viral gene expression upon entry since UL138 is reported to be absent from viral particles [12]. Since these wt viral gene products are expressed early after infection, they may directly or indirectly inhibit MIE transcription. However, using a luciferase activity assay, it was shown that UL138 had no effect on MIE promoter activity [12]. At higher MOIs than used in this report, deletion of UL138 or UL133–138b did not affect MIE protein steady state levels [31], which is in contrast to our results using a lower MOI. The differences in their results may be due to the virion-associated factors like pp71 and gB stimulating MIE transcription at the higher MOIs.

The inhibition of MIE gene expression may explain why clinical viruses containing the UL133 to UL138 gene cluster establish latency in CD34+ cells more efficiently than the laboratory strains [12]. However, the UL133 to UL138 gene cluster increases efficient viral replication in endothelial cells in culture [16]. One explanation for these paradoxical results is that the UL133 to UL138 gene cluster may modulate the cell-type specific outcome of infection [16]. The higher cell-free infectious virus production with the mutant recombinant viruses compared to the wt viruses supports the possibility that the wt viral proteins also suppress egress while not being virion-associated. The location of these viral proteins in the trans Golgi apparatus at late times after infection would support this possibility [12]. Therefore, the wt viral proteins of the UL133 to UL138 gene cluster have a dual role in keeping viral replication suppressed in HFF cells. The mechanism of how these viral proteins from the UL133 to UL138 gene cluster accomplish control of virus production is important to investigate for understanding latency and persistent infection by HCMV.

## Supporting Information

S1 Fig Effect of deletion and stop codon mutation on corresponding viral gene expression. HFF cells were infected with mutant recombinant FIX viruses at an MOI of 1 PFU/cell. Cells were harvested for protein expression at 36 h p.i. Western blot analysis was performed using polyclonal anti-UL133 and anti-UL135 antibody as described in the Materials and Methods. GAPDH served as a loading control.
(TIF)

S2 Fig Viral gene deletion and mutation do not alter the relative amount of pp71 antigen. HFF cells were infected with wt and mutant recombinant FIX viruses at an MOI of 0.5 PFU/cell. Cells were harvested at 3 h p.i. (A) Western blot analysis was performed using monoclonal anti-pp71 antibody as described in the Materials and Methods. GAPDH served as a loading control. (B) Ratio of pp71 and pp65 normalized to GAPDH for two biologically separate experiments analyzed by three independent Western blots.
(TIF)

S3 Fig Effect of viral gene deletion and mutation on IE1-p72 antigen expression at an early time point. HFF cells were infected with wt and mutant recombinant viruses at an MOI of 0.5 PFU/cell. Cells were harvested for immunofluorescence assay of IE1-p72 antigen at 24 h p.i. Nuclei were visualized using 4′,6′-diamidino-2-phenylindole (DAPI).
(TIF)
