# Supplementary figures and images for: The Human Cytomegalovirus UL133-138 Gene Locus Attenuates the Lytic Viral Cycle in Fibroblasts
Source: PLoS One. 2015 Mar 23;10(3):e0120946. doi: 10.1371/journal.pone.0120946 (PMC4370700; doi:10.1371/journal.pone.0120946)

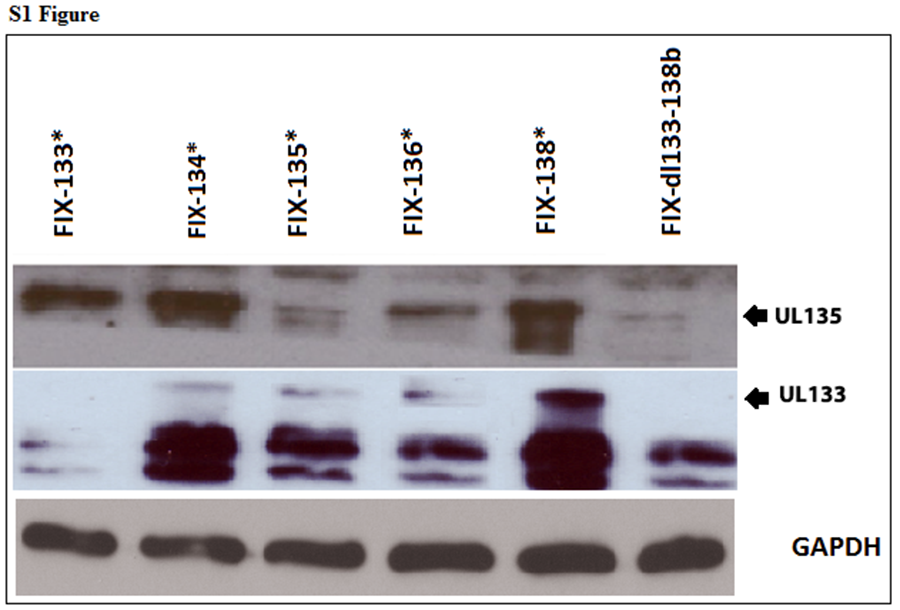

Supplement: S1 Fig — HFF cells were infected with mutant recombinant FIX viruses at an MOI of 1 PFU/cell. Cells were harvested for protein expression at 36 h p.i. Western blot analysis was performed using polyclonal anti-UL133 and anti-UL135 antibody as described in the Materials and Methods. GAPDH served as a loading control. (TIF) [file pone.0120946.s001.tif]

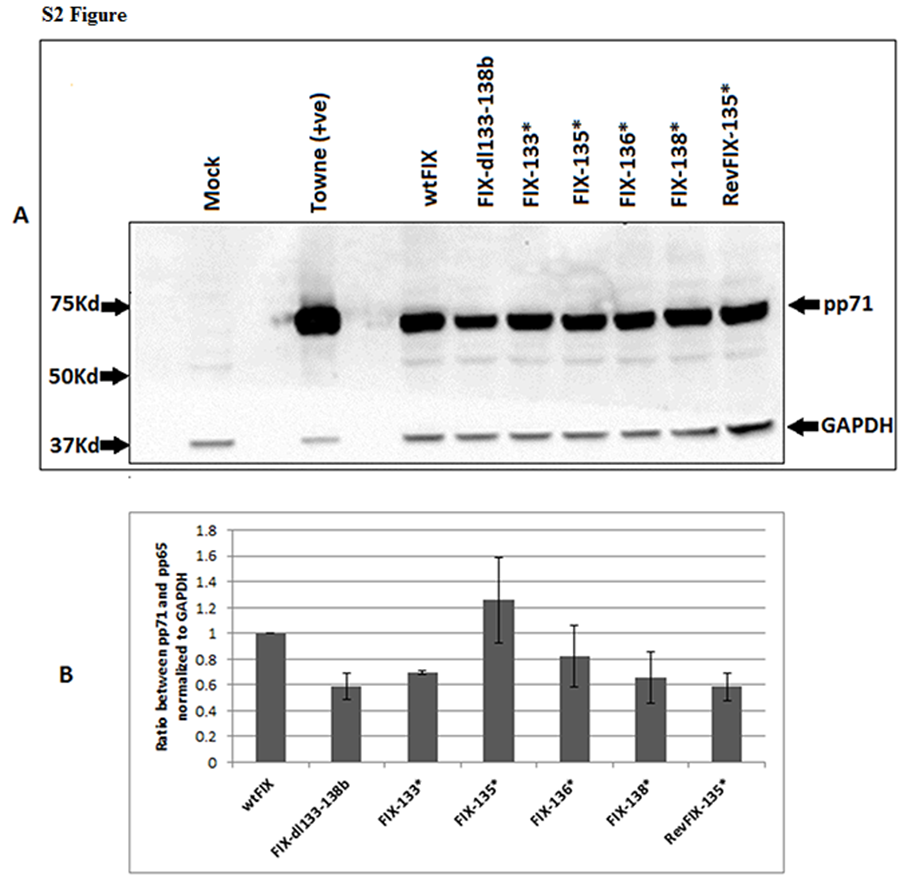

Supplement: S2 Fig — HFF cells were infected with wt and mutant recombinant FIX viruses at an MOI of 0.5 PFU/cell. Cells were harvested at 3 h p.i. (A) Western blot analysis was performed using monoclonal anti-pp71 antibody as described in the Materials and Methods. GAPDH served as a loading control. (B) Ratio of pp71 and pp65 normalized to GAPDH for two biologically separate experiments analyzed by three independent Western blots. (TIF) [file pone.0120946.s002.tif]

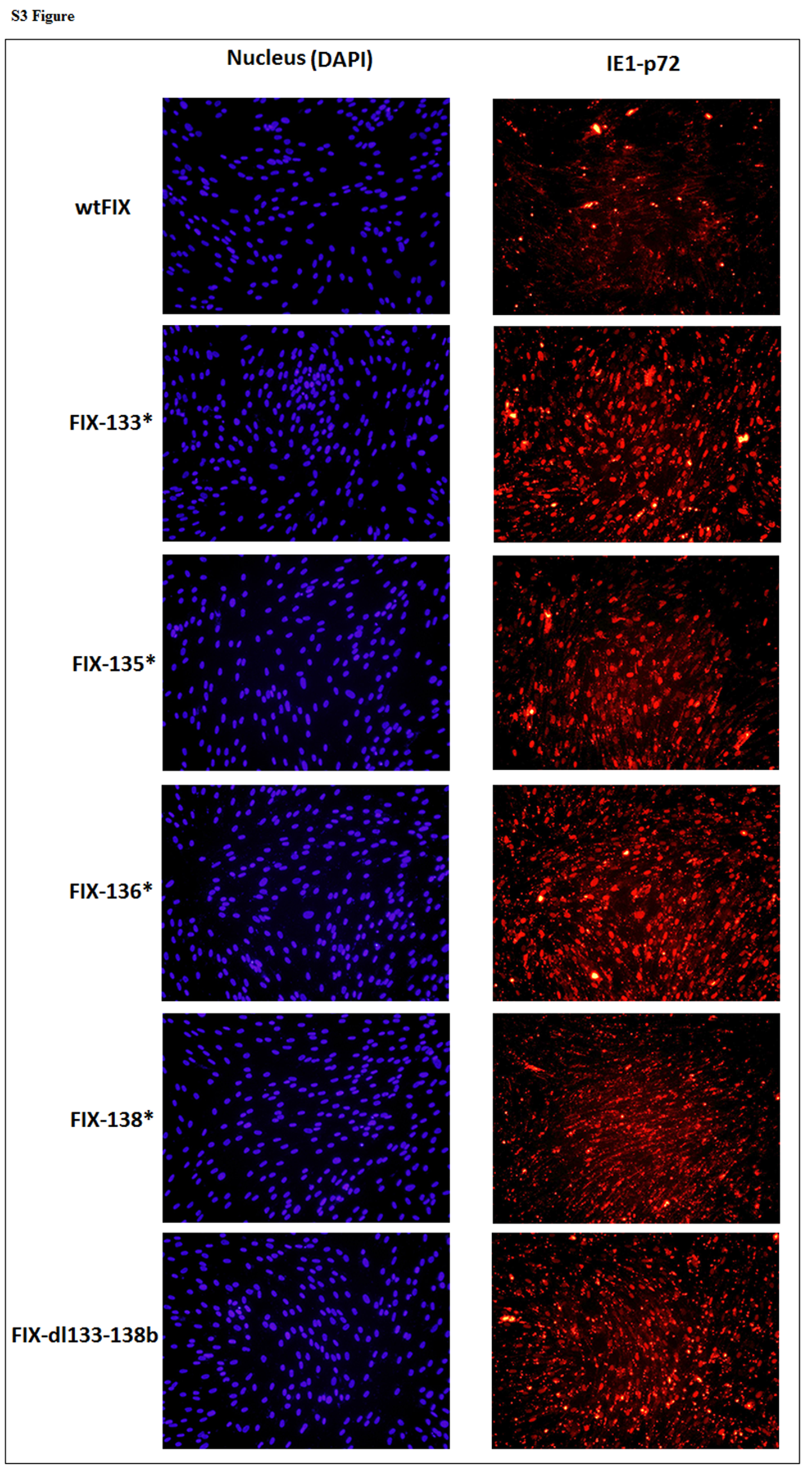

Supplement: S3 Fig — HFF cells were infected with wt and mutant recombinant viruses at an MOI of 0.5 PFU/cell. Cells were harvested for immunofluorescence assay of IE1-p72 antigen at 24 h p.i. Nuclei were visualized using 4′,6′-diamidino-2-phenylindole (DAPI). (TIF) [file pone.0120946.s003.tif]
